# Hexagonal zinc oxide nanoparticles: a novel approach to combat multidrug-resistant *Enterococcus faecalis* biofilms in feline urinary tract infections

**DOI:** 10.3389/fcimb.2024.1505469

**Published:** 2025-01-24

**Authors:** Alaa H. Sewid, Mohamed Sharaf, Azza S. El-Demerdash, Sherif M. Ragab, Fatimah O. Al-Otibi, Mohamed Taha Yassin, Chen-Guang Liu

**Affiliations:** ^1^ Department of Microbiology, Faculty of Veterinary Medicine, Zagazig University, Zagazig, Egypt; ^2^ Department of Forestry, Wildlife and Fisheries, Institute of Agriculture, University of Tennessee, Knoxville, Knoxville, TN, United States; ^3^ Department of Biochemistry and Molecular Biology, College of Marine Life Sciences, Ocean University of China, Qingdao, China; ^4^ Department of Biochemistry, Faculty of Agriculture, AL-Azhar University, Cairo, Egypt; ^5^ Laboratory of Biotechnology, Department of Microbiology, Agricultural Research Center (ARC), Animal Health Research Institute (AHRI), Zagazig, Egypt; ^6^ Department of Botany and Microbiology, College of Science, King Saud University, Riyadh, Saudi Arabia

**Keywords:** *Enterococcus faecalis*, *Moringaoleifera* Lam, hexagonal nanoencapsulation, multi-drug resistance, biofilm

## Abstract

**Introduction:**

*Enterococcus faecalis*, a common inhabitant of the feline gastrointestinal tract, has emerged as a significant pathogen causing urinary tract infections (UTIs) in domestic cats. The rise of multidrug-resistant *E. faecalis* strains and their propensity to form biofilms pose significant challenges in treatment. This study investigated the antibacterial and antibiofilm activities of hexagonal zinc oxide nanoparticles (ZnONPs) alone and in combination with streptomycin and Moringa oleifera leaf extract (MOLe) against multidrug-resistant *E. faecalis* isolates from feline UTIs.

**Methods:**

Antimicrobial susceptibility testing was performed using the Kirby-Bauer disk diffusion method. Biofilm formation was assessed using the crystal violet assay, and biofilm-associated genes (*sprE*, *gel*E, *fsr*ABC) were detected by PCR. ZnONPs, Str/ZnONPs (streptomycin-loaded ZnONPs), and Str/MOLe@ZnONPs (streptomycin and MOLe-loaded ZnONPs) were characterized using FTIR, DLS, TEM, and SEM. The antibacterial and antibiofilm activities of the synthesized nanoparticles were evaluated through time-kill assays, well diffusion assays, and gene expression analysis.

**Results:**

A high prevalence of multidrug resistance was observed among the *E. faecalis* isolates, with significant resistance to ampicillin, vancomycin, and streptomycin. Characterization studies revealed the successful encapsulation of streptomycin and MOLe within the ZnONPs.*In vitro* assays demonstrated that Str/MOLe@ZnONPs exhibited potent antibacterial and antibiofilm activities against the tested *E. faecalis* strains, significantly reducing bacterial growth and biofilm formation.

**Discussion:**

The emergence of multidrug-resistant *E. faecalis* strains necessitates the development of novel therapeutic strategies. This study demonstrates the promising potential of ZnONPs, particularly those loaded with streptomycin and MOLe, in combating biofilm-forming *E. faecalis*. The synergistic effects of the combined formulation may offer a novel approach to overcome antibiotic resistance and improve the treatment outcomes of *E. faecalis* UTIs in domestic cats.

## Graphical Abstract

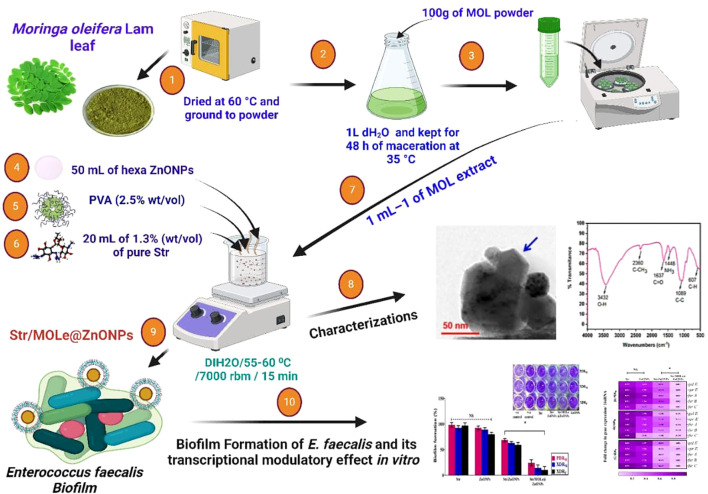

## 1 Introduction

Enterococci are widespread bacteria that are causally to the development of severe urinary tract infections (Weese et al., 2011; Kajihara et al., 2015). Enterococci are often found in cats, and dogs serve as a reservoir for transmitting these bacteria to people (Kataoka et al., 2014; Jackson et al., 2009; Ossiprandi and Zerbini, 2015). The excessive and disorganized use of antibacterial medication has resulted in a rise of drug-resistant *E. faecalis* strains, rendering most medications ineffective against this bacterium (Oguttu et al., 2021). Bacteria acquire resistance to aminoglycosides because their cell wall has insufficient permeability, making these antibiotics ineffective when used individually (García-Solache and Rice, 2019). Furthermore, the ability of these bacteria to form biofilms makes bacteria more resistant to antibiotics (Pereira et al., 2014). Therefore, there is an urgency for developing new anti-enterococcal therapeutic drugs.

*Moringaoleifera* Lam (MOL) is rich in nutrient components, such as essential amino acids, oleic acids, vitamins, and minerals. MOL is well-known for its uses in medicine, including the treatment of a variety of illnesses, the control of the immunological system, and the manifestation of anti-oxidant, anti-diabetic, and anti-cancer qualities (Dhakad et al., 2019). MOL is a plant that may be consumed as a vegetable, and it also has the potential to be used as a medication to cure a broad variety of diseases (Xiao et al., 2020). The extracts of MOL have been observed to contain a phytochemical composition which includes alkaloids, flavonoids, glycosides, tannins, triterpenoids, and steroids (El-Mohamedy and Abdalla, 2014). Numerous studies have shown that various extracts from various tissues of MOL demonstrated antibacterial activities against both gram-negative and gram-positive bacteria, for example, fresh leaf juice against *Pseudomonas aeruginosa* and *Staphylococcus aureus* (Ahmed et al., 2023).

Nanoparticles (NPs) are increasingly used as an alternative to antibiotics (Wang et al., 2017; Ali et al., 2023; El-Demerdash et al., 2023b; Abd El-Emam et al., 2024), as zinc oxide (ZnO) showed antimicrobial activity (Raghunath and Perumal, 2017; Dawwam et al., 2022), against both Gram-positive (Guo et al., 2015), and Gram-negative bacteria (Liu et al., 2009; Reddy et al., 2014; Guo et al., 2015).Recent studies have shown progress in the combination use of antibiotics with nanoparticles, resulting in a synergistic impact. Research on the mechanism of action of combining nanoparticles (NPs) with antibiotics indicates that the increased antibacterial effect may result from a chemical interaction between the NPs and antibiotics. Enhancing antibiotics using nanoparticles decreases the need for high medication doses, lowers toxicity to human cells, and restores their capacity to combat resistant microorganisms (Selvaraj et al., 2019). Nanoparticles combined with antibiotics have boosted the amount of antibiotics at the site where bacteria and antibiotics interact. This combination has improved the binding of antibiotics to bacteria and prevented bacterial efflux pumps from working, resulting in a more effective conjugation. The nanoparticle-antibiotic combination is intended as a substitute for resistant bacteria. Nano-conjugates show a significant enhancement in their biological effectiveness compared to unbound antibiotic molecules (Allahverdiyev et al., 2011). Previous studies have shown an advantageous enhancement in the antibacterial efficacy of streptomycin when paired with nanoparticles, with improvements ranging from(30–87.5%) (Nishanthi et al., 2019; Salar et al., 2015; Aremu et al., 2021).Furthermore, the combination of ZnONPs with antibiotics demonstrated a potential approach that significantly altered the resistance characteristics of multidrug-resistant *P. aeruginosa* strains (El-Telbany et al., 2022), and showed an antibiofilm effect against *S. aureus* (Abdelghafar et al., 2022).

Recent studies have shown that targeting quorum-sensing genes, such as *fsr*, could be a promising approach for developing new anti-enterococcal therapies (Tang et al., 2015; Desouky et al., 2013; Singh and Nakayama, 2014; Shojima and Nakayama, 2014). Additionally, ZnONPs have been demonstrated to inhibit quorum-sensing in various bacteria, including *P. aeruginosa* (Ali et al., 2020; El-Telbany et al., 2022), *S.aureus* (Abdelghafar et al., 2022), and *C. violaceum* (Kamli et al., 2021). This study aimed to evaluate the antimicrobial and antibiofilm activities of hexagonal ZnONPs synthesized using Moringa oleifera leaf extract and loaded with streptomycin against *E. faecalis* isolated from urinary tract infections in pet cats.

## 2 Materials and methods

### 2.1 Samples, isolation and identification of *E. faecalis*

A total of 100 pet cats (50 male, and 50 female), were chosen for urine sample collection at the Animal Health Research Institute in Zagazig, Egypt. The cats had urinary clinical symptoms such as inappropriate urination, extensive licking of the genital region, and frequent and/or protracted efforts to pee. Urine samples were immediately delivered to the laboratory for culture and antibiotic susceptibility testing within 1 hour and kept in cold boxes. The study was conducted following the Declaration of Helsinki by the World Medical Association and was approved by the Research Ethics Committee of the Faculty of Veterinary Medicine, Zagazig University (approval number ZU-IACUC/2/F/215/2023). *E. faecalis* strains were routinely cultivated on Bile EsculinAzide Agar (Thermo Scientific., L and smear, The Netherlands) (Ew and Microbiology, 1997). All presumptive *E. faecalis* isolates with typical black to brown colonies were confirmed by *E. faecalis* primers targeted by the *ddl* gene as described previously (Dutka-Malen et al., 1995).

### 2.2 Antimicrobial susceptibility testing (Kamli et al.)

Antimicrobial susceptibility was evaluated on Mueller–Hinton agar using the disc diffusion method following CLSI guidelines (CLSI, 2023). Examined were 11 standard antimicrobial discs from Oxoid, Cambridge, UK, representing 8 antimicrobial categories, including aminoglycosides [streptomycin (S; 10 µg), carbapenemes[Imipenem (IMP;10 µg),andmeropenem (MEM; 10 µg)], Fluoroquinolones [Levofloxacin(LEV; 5 µg) and ciprofloxacin (CIP; 5 µg)],glycopeptide [Vancomycin (VA;30 µg), and Teicoplanin (TEC;30 µg)], glycylcycline[Tigecycline (TGC;15 µg)],penicillins [ampicillin (AM; 10 µg)], oxazolidones [lenzolid (LNZ; 30 µg)], and tetracyclines [doxycycline (DO; 30 µg)]. The multiple antimicrobial resistance (MAR)indexes were computed as previously described (Tambekar et al., 2006). Pandrug-resistance refers to resistance to all antimicrobial agents, extensive drug resistance is resistance to all classes of antimicrobial agents except 2 or fewer, and multidrug-resistance indicates resistance to three or more classes of antimicrobial agents, as detailed in previous reports (Magiorakos et al., 2012).

### 2.3 Biofilm formation, and detection of the *gelE*, *sprE*, and *fsr (A, B, C)* genes by conventional polymerase chain reaction

All *E. faecalis* isolates were checked for biofilm production using crystal violet assay and compared with the control strain (*E. faecalis*ATCC 29212,and *E. faecalis*ATCC 51299) as previously described (Kafil et al., 2013; Mathur et al., 2006).The optical density (OD) was measured at a wavelength of 570nm. The isolates were classified into several categories based on their OD values: strong biofilm formers (OD570> 2), medium biofilm formers (OD570 > 1 but <2), weak biofilm formers (OD570> 0.5 but <1), and non-biofilm formers (OD570 ≤ 0.5) (Mohamed et al., 2004; El-Demerdash et al., 2023b). *E. faecalis* isolates underwent DNA extraction using the QIAamp DNA Mini kit from Qiagen, Gmbh, and Hilden, Germany, following the manufacturer’s instructions. The isolates were analyzed for the presence of gelatinase *(gelE)*, serine protease *(sprE)* and quorum-sensing for gene locus (*fsr; A, B, C*) using previously designed primers (Dutka-Malen et al., 1995; Popović et al., 2018; Nakayama et al., 2002).The PCR assays used DNA from *E. faecalis* ATCC 29212 and *E. faecalis* ATCC 51299, with sterile saline used as positive and negative controls, respectively.

### 2.4 Extraction of biochemical compounds of MOLe

Briefly, the leaves of *moringa oleifera* leaves were washed with distilled water, dried, and ground into a fine powder. 100 g of the powder was soaked in 1000 mL of distilled water for 48 hours at 35°C. The mixture was then filtered, and the supernatant was obtained after centrifugation at 9,000xg for 10 minutes. The supernatant was diluted for foliar application.

### 2.5 Synthesis of exo-capsulation hexagonal Str/MOLe@ZnONPs

#### 2.5.1 Deposition of hexagonal ZnONPs

ZnONPs were synthesized using a hydrothermal technique including Zn(NO3)2·6H2O and NaOH precursors, as outlined in the published work (Fiedot-Tobola et al., 2018). A 0.6 M solution of zinc nitrate hexahydrate (Zn(NO3)2·6H2O) in aqueous ethanol was stirred continuously for 45 minutes to dissolve the salt. Similarly, a 1 M aqueous ethanol solution of NaOH was prepared. The zinc nitrate hexahydrate solution was then slowly added to the NaOH solution dropwise over 25 minutes while stirring vigorously. The reaction mixture was stirred for 1 hour at a constant temperature. The precipitate was allowed to settle overnight and then separated by centrifugation. The zinc oxide nanoparticles were washed four times with distilled water and ethanol and dried in an air atmosphere at 90°C.

#### 2.5.2 Str-coated hexagonal ZnONPs

A solution of PVA at a concentration (2.5% wt/vol) was applied onto 50 ml of hexa ZnONPs while being vigorously stirred with a powerful magnetic at 1000 rpm for 15 h at 25 to 27°C and a pH of 7.2. This process followed the emulsion-coacervation technique (Sharaf et al., 2022; Hu et al., 2006),with some alterations. Next, 20 mL of 1.3% (wt/vol) solution of pure Str in deionized water was agitated for 10 min and then poured over PVA-ZnONPs. The mixture was then rapidly swirled for 15 min at the same temperature as before. The excess uncoated PVA polymers and any other medications were eliminated from the Str/ZnONPs using several rounds of centrifugation(Centrifuge 5427 R) at 7,000 rpm for 15 min, each time with additional ddH_2_O, followed by drying in a vacuum for 24 h 55-60°C.

#### 2.5.3 Exo-capsulation hexagonal Str/MOLe@ZnONPs

About 0.2 mg of Str/ZnONPs were dissolved in 30 mL–1 of dH2O at 70-80°C for 30 min. 0.5ml of tween 20 was added to the mixture, which was then agitated at 300 rbm for 15 min at 50°C to preactivate lignin. 1 mL of MOLe at a concentration of 1mg mL–1 was added drop by drop and subjected to a homogenizer (Heidolph, DIAX 900) for 10 min at 30000 rpm. Subsequently, a high-intensity ultrasonic horn (Hielscher Ultrasound Homogenizer UIP1000, 20 kHz, 50% amplitude, Ti-horn) was used for 1 hour at 50°C to create the Str/MOLe@ZnONPs. The mixture was centrifuged at 18,000 rpm for 20 min to remove unreacted molecules in the supernatant. The particle was concentrated fivefold in deionized water before being resuspended. Subsequently, low-intensity ultrasound was used to disperse the particles. The NPs were finally centrifuged at 500g for 10 min to eliminate bigger aggregates. The Str/MOLe@ZnONPs were kept at 4°C.

### 2.6 Characterizations of exo-capsulation hexagonal Str/MOLe@ZnONPs

DLS was used to assess formulation particle size and ζ-potential charge means (Malvern Instruments, UK). 3 ml of bareZnONPs, MOLe@ZnONPs and Str/MOLe@ZnONPs were diluted in deionized water, put in a cell cuvette, and measured four times to estimate the size, and three ζ-potential charge the mean ± SD (Sharaf et al., 2021). The optical properties of the nanopowders (ZnONPs, MOLe@ZnONPs, and Str/MOLe@ZnONPs) were analyzed using Fourier transform infrared (FTIR) spectroscopy in KBr pellets. The particle size and morphology of the samples were characterized using scanning electron microscopy (SEM) and high-resolution transmission electron microscopy (TEM) (JCM-7000 Neo Scope™ Benchtop TEM; JEOL, Japan).

### 2.7 Antibacterial activities

#### 2.7.1 Agar well diffusion assay

The antibacterial effects of Str/MOLe@ZnONPs, Str/ZnONPs, and MOLe@ZnONPs were evaluated against *E. faecalis* isolates with high antimicrobial resistance profiles (PDR[isolate code 21], and XDR [isolate code 32,and 34]) and strong biofilm production. The results were compared to streptomycin discs (10 µg, Oxoid, Cambridge, UK). Bacterial suspensions in sterile saline were adjusted to a concentration of 1.5 × 108 CFU/mL and cultured on Mueller–Hinton (MH) agar. Wells were created in the agar plates, and 100 µL of each chemical was added to each well. Sterile water served as the negative control. The plates were incubated at 37°C for 24 hours, and the zones of growth inhibition were measured to assess the antimicrobial effectiveness of the tested drugs (Wang et al., 2010).

#### 2.7.2 Minimum inhibitory concentration, and minimum bactericidal concentration

The minimal inhibitory concentration was identified using microbroth dilution techniques. The concentrations of Str/MOLe@ZnONPs, Str/ZnONPs, MOLe@ZnONPs, and Str were diluted in a two-fold manner from 1 to 1024 μg/mL. They were then mixed with a standardized inoculum solution and incubated at 37°C for 24 h. The Minimum Inhibitory Concentration (MIC) was defined as the lowest concentration at which no visible growth was observed. The Minimum Bactericidal Concentration (MBC) was determined by culturing 10 μL of each clear well on Mueller-Hinton agar plates and identifying the lowest concentration that resulted in a 99.9% reduction in the initial inoculum after overnight incubation (Baek and An, 2011). The tolerance levels of *E. faecalis* against Str/MOLe@ZnONPs, Str/ZnONPs, MOLe@ZnONPs, and Strwere assessed using a specific formula: Tolerance = MBC/MIC as previously described (May et al., 1998).

#### 2.7.3 Time-kill assay

A single colony of *E. faecalis* was cultured in BHI broth at 37°C overnight. The bacterial cells were then collected by centrifugation at 6000 x g for 10 minutes and resuspended in the lowest inhibitory concentration of Str/MOLe@ZnONPs, Str/ZnONPs, MOLe@ZnONPs, and Str. At 0, 2, 4, 8, and 24 h, samples were collected, and colonies were counted. The number of viable cells was determined as colony-forming units per mL (CFU/mL) using the drop plate technique with a 10-fold serial dilution in saline. The diluted solution was then dropped into MHA plates and incubated at 37°C for 24 h (Tan et al., 2019).

### 2.7.4 Biofilm formation

Subinhibitory concentrations (1/2 × MIC) of Str/MOLe@ZnONPs, Str/ZnONPs, MOLe@ZnONPs, and Str, along with an antibiotic-free medium as a negative control, were used to evaluate their effect on *E. faecalis* formation. Biofilms were measured by crystal violet testing, according to established protocols (Mathur et al., 2006). The optical density of the biofilm mass was quantified using a microplate ELISA reader (Huma Reader HS, Wiesbaden, Germany) at a wavelength of 590 nm. The experiments were conducted in triplicate. Any influence of nanoparticles on the measurement was subtracted from the absorbance caused by the samples. The average result was then presented with ± SD. The biofilm development was assessed in triplicate in separate trials and expressed as the ratio of Str/MOLe@ZnONPs, Str/ZnONPs, MOLe@ZnONPs, and Str compared to the untreated negative control.

### 2.8 Gene expression related to virulence in biofilm culture

qRT-PCR was performed on biofilm cultures treated with sub-inhibitory doses of Str/MOLe@ZnONPs, Str/ZnONPs, MOLe@ZnONPs, and Str, as well as an untreated control. RNA was extracted using the QIAampRNeasy Mini kit (Qiagen) and treated with DNase I to remove genomic DNA. Real-time PCR amplification was performed using HERA SYBR^®^ Green RT-qPCR Master Mix (Willowfort) and novel primers (**Table 1**) designed for the *gelE*,*sprE*, and quorum-sensing *fsr* (A, B, C) virulence genes. Primers were designed using Primer3 and FastPCR software, optimized for specificity and sensitivity using touchdown PCR, and validated experimentally. The *16S rRNA* gene was used as an internal control. PCR conditions were as follows: reverse transcription at 50°C for 30 minutes, followed by 40 cycles of denaturation at 94°C for 15 seconds, annealing at 60°C for 30 seconds, and extension at 72°C for 30 seconds. The relative expression of target genes was calculated using the 2^-ΔΔCt method and normalized to the *16S rRNA* gene (Yuan et al., 2006; Massol-Deya et al., 1995).

**Table 1:** Novel primers for gene expression analysis of biofilm-associated genes in *Enterococcus faecalis* (Syper-green real time PCR assay).

Genes	Primers (5’-3’)	Product size
*16S rRNA*	F: TTCTTTCCTCCCGAGTGCTT R: CTCTCAGGTCGGCTATGCAT	250 bp
*gelE*	F: GGTGCGCCTACATTCAAAGA R: ACCAGGAACATAGCCAGCTT	192bp
*sprE*	F: GATACAACCGAAGCGCCTTT R: ACCAAGCATCATCTTTGGCA	184 bp
*fsrA*	F: CAGGCAGGATTTGAGGTTGC R: GACACATGTTTCGACCTCTTTT	180 bp
*fsrB*	F: TCTTCTGTGAGCTTACCGTTT R: GACCGTAGAGTATTACTGAAGCA	214 bp
*fsrC*	F: TCGCCAGAGATTTCACCTGA R: CGAAACATCGCTAGCTCTTCG	215 bp

### 2.9 Statistical analysis

Each experiment was performed in triplicate, and all results are shown as mean ± standard deviation. Statistical significance was determined using one-way analysis of variance (ANOVA) followed by Tukey’s *post hoc* test (P < 0.05). Analyzes were performed using SAS 9.4 for Windows x64 by the SAS Institute and graphical results were plotted using GraphPad Prism software (version 8, GraphPad Inc. Software).

## 3 Result

### 3.1 Occurrence of *E. faecalis*, and antimicrobial susceptibility patterns

The general prevalence of *E. faecalis* was 14% as reported in **Supplementary Table S1**. 16% of the 50 female samples included *E. faecalis*, whereas 12% of the 50 urine samples from male companion cats had *E. faecalis*.

**Figure 1:**
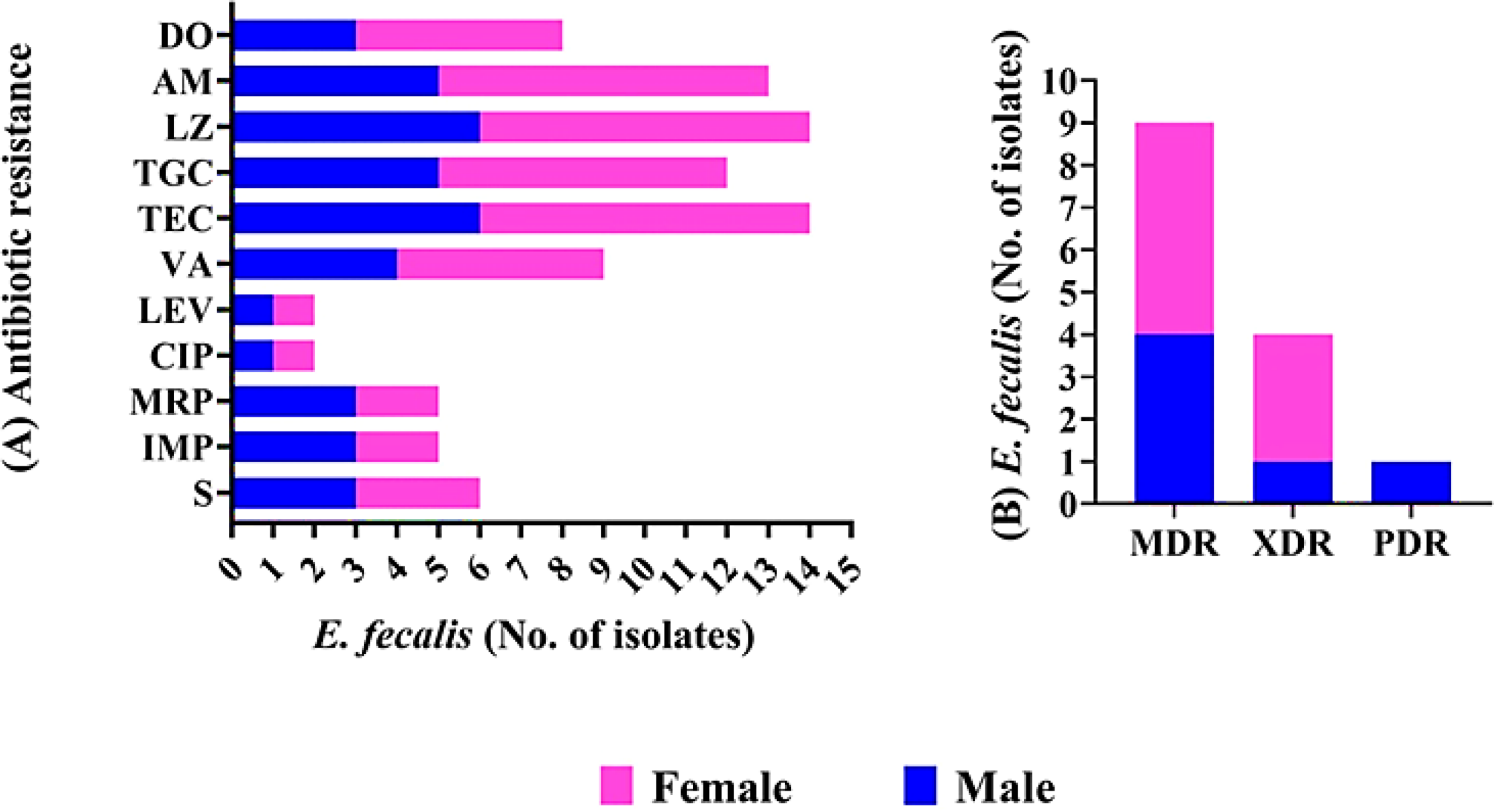
Antimicrobial resistance pattern of *E. fecalis* isolated from female, and male pet cats. **(A)** The patterns of antimicrobial resistance are shown for streptomycin (S), Imipenem (IMP), meropenem (MEM), Levofloxacin (LEV), ciprofloxacin (CIP),Vancomycin (VA), Teicoplanin (TEC), Tigecycline (TGC),ampicillin (AM), lenzolid (LNZ), and doxycycline (DO). **(B)** Presence of MDR, XDR, and PDR classifications in *E. fecalis*.

The antimicrobial susceptibilities of *E. faecalis* isolates were determined *in vitro* (**Supplementary Tables S2, S3**; **Figure 1A**). All isolates were 100% resistant to Teicoplanin and Linezolid. High resistance rates were observed for Tigecycline (87.5% and 83.3%) and Ampicillin (100% and 83.3%) in *E. faecalis* isolates from female and male sources, respectively. Lower resistance rates were detected for both ciprofloxacin and levofloxacin (12.5% and 16.6%) in *E. faecalis* isolates from females and males, respectively. Notably, *E. faecalis* isolates showed high resistance rates to both Imipenem and meropenem, reaching 35.7%. Additionally, 64.2% of isolated *E. faecalis* species were resistant to vancomycin, while 42.8% were resistant to streptomycin.

The investigation of the antibiogram in **Supplementary Table S3** indicated that *E. faecalis* isolates showed resistance to 5–11 antimicrobial drugs, with MARs ranging from 0.45 to 1.00, and displayed 12 unique resistance patterns. PDR, XDR, and MDR patterns were identified in the studied isolates as shown in **Supplementary Table S3**, **Table 2**; **Figure 1B**. None of the isolates examined were completely susceptible to all antibiotics tested. One out of 14 *E. faecalis* isolates showed pan-drug resistance patterns [isolate code 21], being resistant to all antimicrobial drugs tested (7.1%). Four of the isolates tested (28.5%) had XDR characteristics [isolate code 9,11,32,and 34]. However, 9 of *E. faecalis* showed MDR patterns[isolate code 3,5,6,7,10,14,16, 24,and 45] (64.2%). Regarding the isolation source, five, and three *E. faecalis* isolated from female origin exhibited MDR[isolate code 6,10,16,24,and 45] and XDR [isolate code 9,11,and 34] profiles, respectively, while the PDR [isolate code 21] *E. faecalis* isolates originated from male origin.

**Table 2:** The presence of multidrug-resistant (MDR), extensively drug-resistant (XDR), and pandrug-resistant (PDR) categories in *E. faecalis* isolates from female and male pet cats.

Resistance Category	Resistance to Antimicrobial Class (*n* =8)	Resistance to Antimicrobial Agent (*n* = 11)	No. of Resistant *E. fecalis*
			(*n* = 14)
MDR (*n* = 9)	4	5	3 (Male), 1 (Female)
	5	5	1 (Male), 1 (Female)
		6	3 (Female)
XDR (*n* = 4)	6	7	2 (Female)
		8	1 (Female)
	7	9	1 (Male)
PDR (*n* = 1)	8	11	1 (Male)

### 3.2 Biofilm formation and virulence determinants of *E. faecalis* isolates

**Figure 2:**
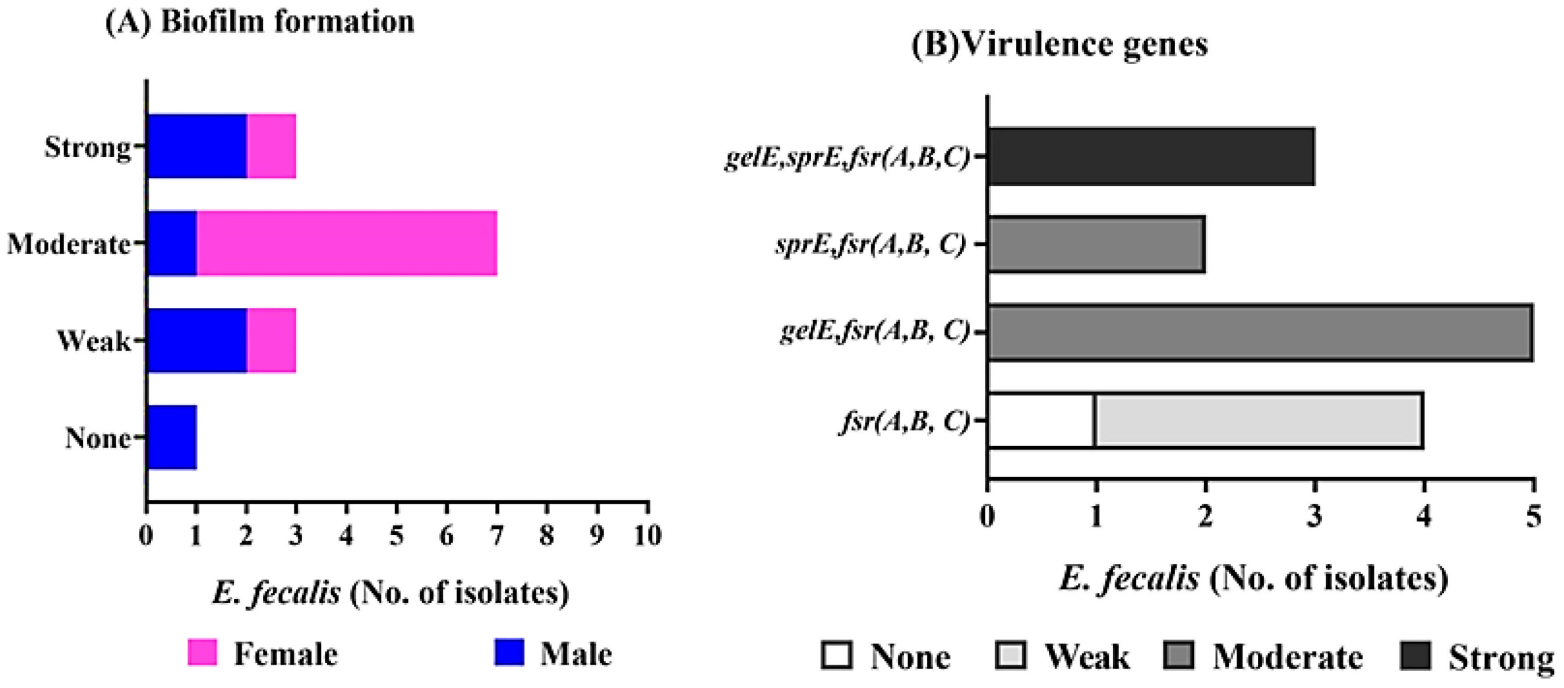
Biofilm formation, and virulence determinant genes of *E. fecalis* isolated from female, and male pet cats. **(A)** Determination of biofilm formation by crystal violet stain showing none, strong, moderate, and weak biofilm producers. **(B)** Detection of the *gelE*, *sprE*, and *fsr (A,B,C)* genes by PCR, and its correlation with biofilm formation.

*E. faecalis* isolates were tested for biofilm formation by using the crystal violet staining method (**Supplementary Table S3**; **Figure 2A**), in which 3(3/14 = 21.4%), and 7(7/14 = 50%) isolates are strong biofilm producer[isolate code 21,32,and 34], and moderate biofilm producer[isolate code 6,7,9,10,16,24,and45] respectively. On the other hand, only one isolate isn`t producing biofilm (1/14 = 7.1%) [isolate code 3], and 3 isolates are weak producer (21.4%) [isolate code 5,11,and 14]. Of note, the overall *E. faecalis* isolated from female origin showed biofilm producer with the highest number exhibited moderate biofilm producer (6/8 = 75%) [isolate code 6,9,10,16,24,and 45].While, most *E. faecalis* isolated from male origin showed strong [isolate code 21, and 32] and weak biofilm producer [isolate code 5,and14] (2 isolate for each). Moreover, two *E. faecalis* isolates from male origin showed moderate [isolate code 7] and none biofilm producer [isolate code 3] (1 isolate for each).

**Figure 3:**
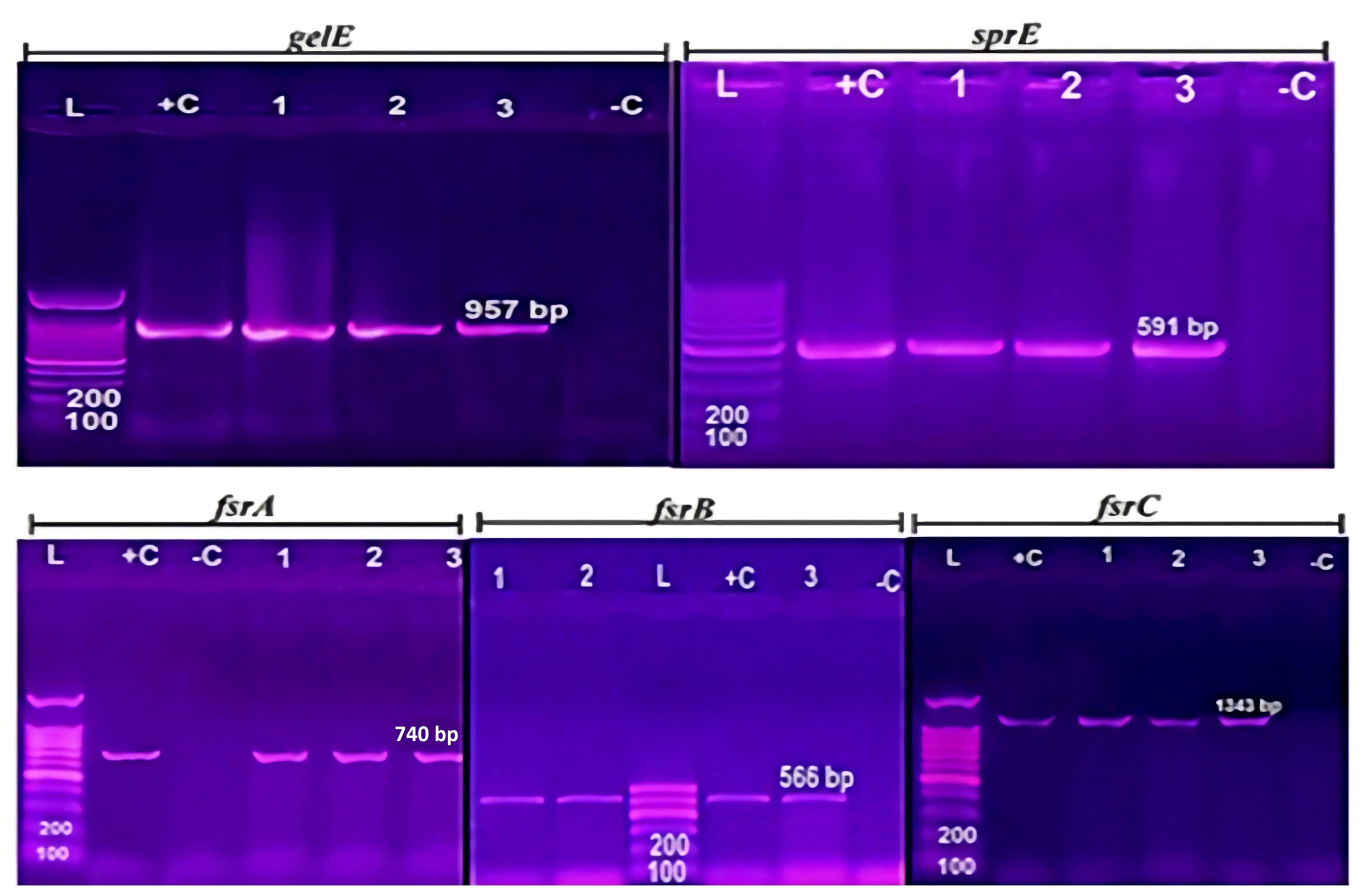
Agarose gel electrophoresis of biofilm genes *gelE*, *sprE*, and *fsr* (A,B,C): DNA ladder (100 bp), control positive and control negative (DNase water) are shown in each lane by L,C+, and C-. The same tested *E. fecalis* isolates [isolate code 21,32, and 34] are shown in 1, 2, 3 lanes. The biofilm genes *gelE*, *sprE*, and *fsr* (A,B,C) of all *E. fecalis* isolates are provided in **Supplementary Table S3**.

PCR analysis was performed to identify genes related to virulence and biofilm formation, including *gelE, sprE*, and *fsrABC* (**Supplementary Table S3**; **Figure 2B**). All 13 biofilm-producing isolates were positive for the *fsrABC* gene locus [isolate code 5,6,7,9,10,11,14,16,21,24,32,34 and 45], resulting in expected DNA fragments of 740, 566, and 1343 bp, respectively (**Figure 3**). The *gelE* gene was detected in 8 (8/13 = 61.5%) biofilm-forming isolates [isolate code 7,9,10,21,24,32,34, and 45], including 3 strong biofilm producers [isolate code 21,32, and 34] and 5 moderate biofilm producers [isolate code 7, 9, 10, 24, and 45]. The *sprE* gene was detected in 5 *E. faecalis* isolates[isolate code 6, 16, 21, 32, and 34], including 3 strong biofilm producers [isolate code 21,32, and 34] and 2 moderate biofilm producers [isolate code 6, and 16]. Isolates lacking *gelE* and *sprE* genes, despite the presence of *fsrABC*, exhibited weak or no biofilm formation [isolate code 3,5,11 and 14].

### 3.3 Characterization of hexagonal zinc oxide nanocapsulation

#### 3.3.1 Surface morphology analysis

**Figure 4:**
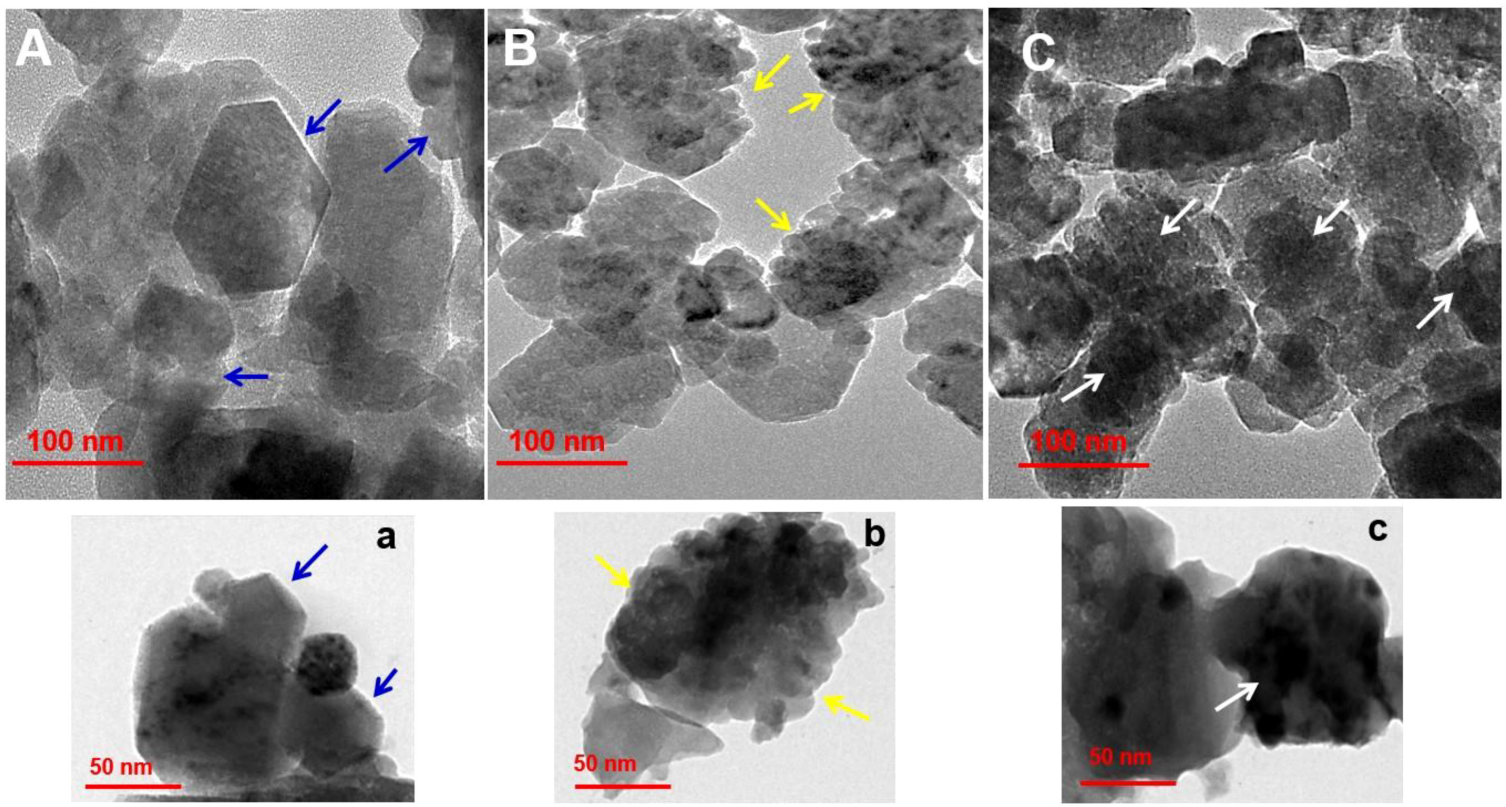
Surface morphology analysis by of **(A-a)** ZnONPs, **(B-b)** Str/ZnONPs and **(C-c)** Str/MOLe@ZnONPs by TEM. Arrow symbol highlights specific features of the nanoparticles.

Size, shape and surface morphological structure of nanoparticles were studied using TEM, the results are shown in **Figures 4A–C** and **4a–c** at 100 and 50 nm, respectively. The produced formulations have varied morphological surfaces, according to TEM. The ZnONPs formed were hexagonal and agglomerated, with an average size ~60 nm (see blue arrow, **Figure 4Aa**). After loading the antibiotic, TEM images were showed several gaps/bumps on the surface of Str/ZnONPs with an average size ~200 nm (see yellow arrow, **Figure 4Bb**), which decreased by a large percentage after coating and capsulating the surface with MOLe with a smooth surface with an average size ~250nm of Str/MOLe@ZnONPs (see white arrow, **Figure 4Cc**). High-resolution transmission electron microscopy (HTEM) (**Supplementary Figure S2A**) and selected area electron diffraction (SAED) pattern in **Supplementary Figure S2B** exhibits concentric rings of bright spots confirming the preferential orientation and highly crystalline nature of ZnONPs. whereas (101), (102), (110), and (103) planes correspond to the wurtzite structure of ZnO. This confirms the formation of NC and corroborates with the FTIR results.

**Figure 5:**
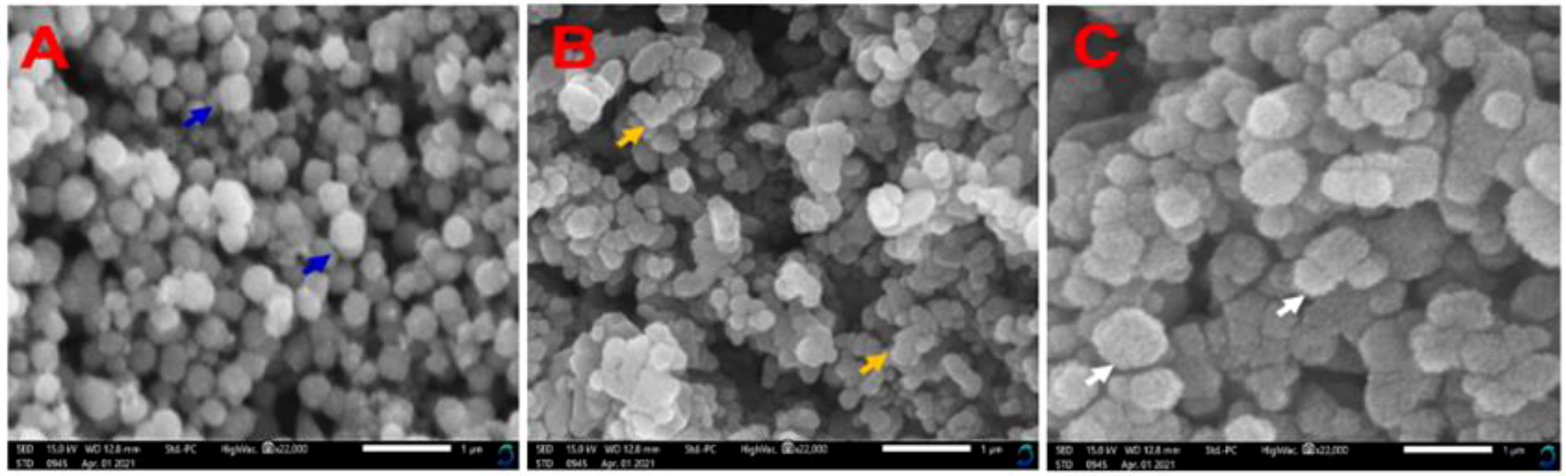
SEM analysis of nanocapsulation synthesized **(A)** ZnONPs, **(B)** Str/ZnONPs and **(C)** Str/MOLe@ZnONPs at Magnification: ×22,000 (1µm). Arrow symbol highlights specific features of the nanoparticles.

Furthermore, the nanoparticles were analyzed by SEM to determine their dimensions, geometry, and surface morphology. The morphologies of ZnONPs are significantly influenced by Str and MOLe. The nanosynthesized formulas have a spherical, hexacrystalline structure of ZnONPs (see blue arrow **Figure 5A**), besides, agglomerated in clusters with rough in appearance of each Str/ZnONPs and Str/MOLe@ZnONPswith an average particle size from 50 to 260 nm, as confirmed by their precise structural properties (see yellow and white arrow **Figures 5B, C**).

#### 3.3.2 DLS

DLS was used to evaluate the particle size (PS), polydispersity index (PDI), and ζ-potential of ZnONPs, Str/ZnONPs, and Str/MOLe@ZnONPs. The average results of all the calculations demonstrated a distribution of PS in nanometers, the findings are included in **Table 3** and visually shown in **Supplementary Figure S1**. The results obtained from DLS analysis indicated that the nanoparticles had average diameters of around 65.9 ± 3.57nm,199.3 ± 12.93nm and 271.4 ± 19.10nm, with corresponding PDI of 0.326 ± 0.037, 0.690± 0.12and 0.142 ± 0.05for ZnONPs, Str/ZnONPs, and Str/MOLe@ZnONPs, respectively (**Supplementary Figures S1A, C**). The results indicated that the incorporation of Str and the application of a MOLe coating led to an augmentation in the dimensions of ZnONPs. The ζ-potential values of nanoparticles are often used to assess their stability. In this study, the ζ-potential values of ZnONPs, Str/ZnONPs, and Str/MOLe@ZnONPs were found to be –19.8± 0.22mV,–29.1± 0.12, and –33.1 ± 0.55mV, as shown in (**Figures 5A–C**). Row Coloration Data (RCD) was clarified tree formulations by three variables (size, PDI and ζ-potential) where the first and second component were (94.9 - 2.2%), (96.3-1.9%) and (97.3-3.9%) of ZnONPs, Str/ZnONPs, and Str/MOLe@ZnONPs the total variance (99.2%) during analysis (10^7^ µs), as score plot carve (**Supplementary Figure S1G–I**).

**Table 3:** Characteristics the mean size (Z-average), polydispersity index (PDI), ξ-potential to different nano-formulation.

Formulation	Size	PDI	ξ-potential (mv)
ZnONPs	65.9 ± 3.57	0.326± 0.037	–19.8± 0.22
Str/ZnONPs	199.3± 12.93	0.690± 0.12	–29.1± 0.12
Str/MOLe@ZnONPs	271.4 ± 19.10	0.142± 0.05	–33.1 ± 0.55

Results are expressed as mean ± standard deviation (mean ± SD) *n*=3.

#### 3.3.3 FTIR

**Figure 6:**
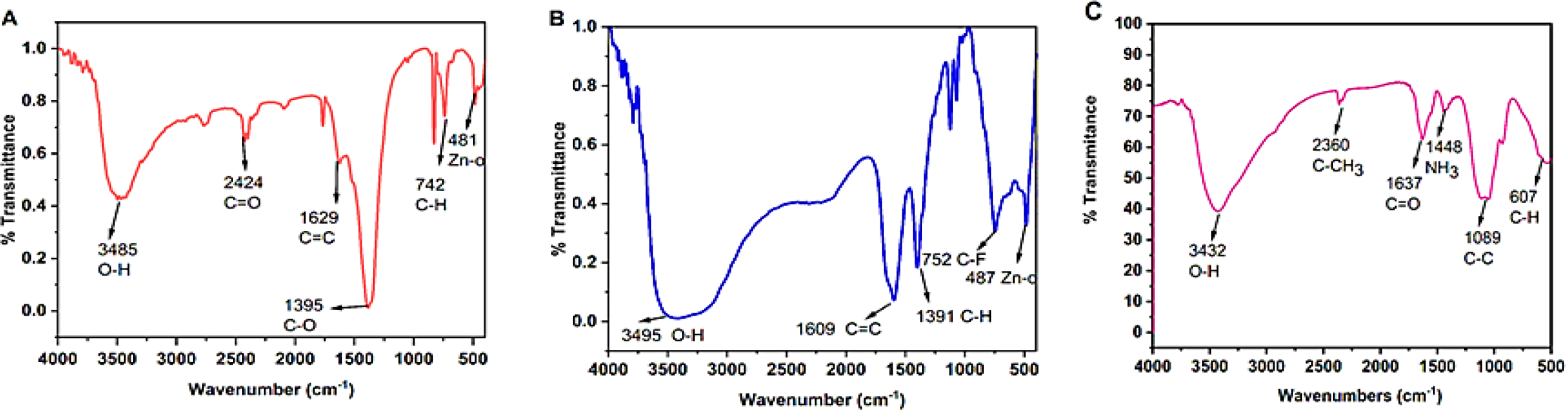
FTIR spectra analyzed the synthesis of **(A)** ZnONPs, **(B)** Str/ZnONPs and **(C)** Str/MOLe@ZnONPs.

FTIR analysis is a successful method for illustrating the chemical composition of synthesized substances. The FTIR spectra were analyzed at a scan range of 4000 –500 cm^-1^ to determine the functional groups that were involved in the synthesis of ZnONPs, Str/ZnONPs and Str/MOLe@ZnONPs were depicted in **Figure 6**. In this investigation, FTIR spectrum of synthesized ZnONPs showed the peak at 481 cm^-1^ corresponds to the absorption of the Zn–O bond, the bands at 3485, 2424, 1629, 1395, and 742 cm^-1^ correspond to the bonds O–H, C = O, C = C, C–O, and C–H, respectively, as shown in **Figures 6A, B** showed the greatest peaks in the FTIR spectrum of Str/ZnONPs at 3495 cm^-1^ correspond to O–H stretching vibrations of phenols, The FTIR spectral bands at 1609 cm^-1^ is assigned to –C=C stretching of functional group, the maximum at 1399 cm^-1^ corresponds to C-H stretching vibrations of the alkene group. Other brief peaks between 500 and 800 cm^-1^ have been ascribed to the presence of metal–oxygen (Zn–O). However, **Figure 6C** showed the FTIR absorption spectra of biosynthesized, vacuum-dried Str/MOLe@ZnONPs revealed the presence of bonds due to O–H stretching (around 3,432 cm^-1^) and C–CH_3_ stretching (around 2,360 cm^-1^). The FTIR spectrum revealed a C=O-corresponding absorption band at 1,637 cm^-1^. The bands seen at 1448 cm^-1^ are attributed to the modes of vibration of the amino group (NH_3_). A band at 1089 cm^-1^indicated C=C elongation. The bands evident between 500 and 650 cm^-1^ denoted the presence of the R–CH group. The FTIR spectra of synthetic *M. oleifera* leaf extract are shown in (**Supplementary Figure S3A**), the highest FT-IR peaks at 3490 cm1 in the MOLe FTIR spectrum are attributed to the O–H stretching vibrations of phenols. The peak observed at 2090 cm^-1^ corresponds to the C-H stretching and bending vibrations. At 1670 cm^-1^, the active C=O stretching vibrations. The FTIR spectrum of streptomycin (Str) was observed to contain absorption bands at 3345, 2180, 1451, 1030, and 850 cm^-1^ due to the N-H in the amino group, C-H, CH_2_ group, C–N stretching, and C–C group, respectively (**Supplementary Figure S3B**).

### 3.4 Antimicrobial activities

#### 3.4.1 Antibacterial activity against planktonic *E. faecalis* cells

**Figure 7:**
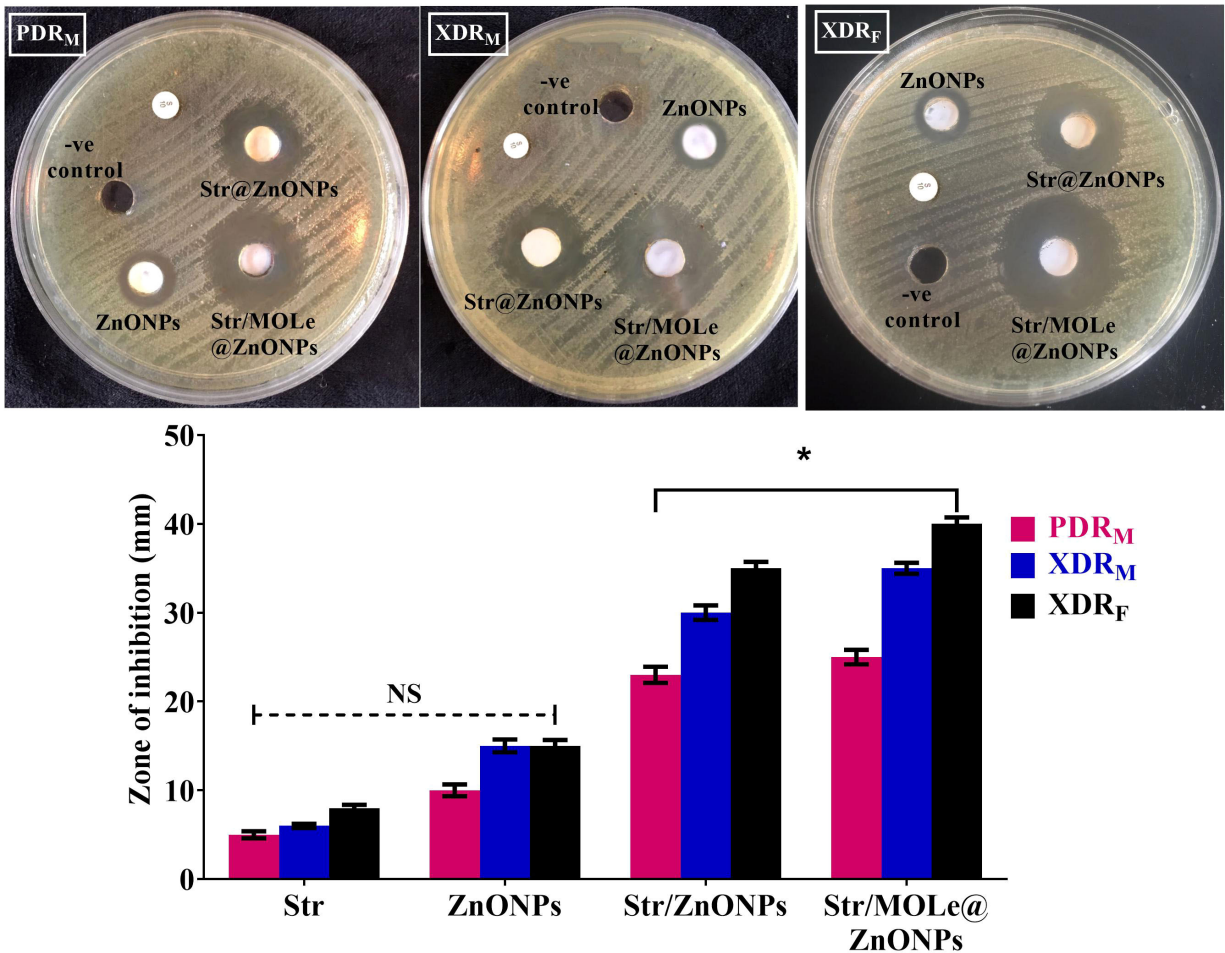
The inhibition zone of ZnONPs, Str/ZnONPs and Str/MOLe@ZnONPs compared with Str were determined by *in vitro* of *E. fecalis* bacterial growth by agar well diffusion assay; extensively drug-resistant isolate of female origin (XDRF; isolate code 34), extensively drug-resistant isolate of male origin (XDRM; isolate code 32), and pandrug-resistant isolate of male origin (PDRM; isolate code 21). Each column represents the average ± standard deviation of three separate studies along with illustrative visuals. *Signifies statistically significant differences (*P*< 0.05), whereas NS indicates non-significant differences (*P*> 0.05) in comparison to the control group.

Three *E. faecalis* isolates, two categorized as extensively drug-resistant (XDR) [isolate code 32, and 34] and one as pan drug-resistant (PDR) [isolate code 21], originating from both male (2 isolates) [isolate code 21, and 32] and female (1 isolate) [isolate code 34] sources, were tested for susceptibility to Str/MOLe@ZnONPs, Str/ZnONPs, and MOLe@ZnONPs. Their resistance to at least 7 antimicrobial agents and strong biofilm production were considered. The evaluation included measuring inhibition zone diameters and determining minimum inhibitory concentration (MIC) values (**Figure 7**; **Supplementary Table S4**). The findings indicated a notable difference in the inhibitory zone size among Str/MOLe@ZnONPs, Str/ZnONPs, streptomycin, and unloaded MOLe@ZnONPs (p<0.05). The Str/MOLe@ZnONPs exhibited the highest antibacterial activity, with an inhibition zone diameter of up to 40mm (**Figure 7**). This efficacy was reflected in the low recorded MICs (≤ 16 μg/mL) (**Supplementary Table S4**) compared with 35 mm diameter inhibition zones of Str/ZnONPs, and MICs (≤ 32 μg/mL). Furthermore, the MBC values of Strand Str/MOLe@ZnONPs were two-fold higher than MIC values with a tolerance equal to 2 indicating their bactericidal effect. While, both of MOLe@ZnONPs, and Str antibiotic alone didn`t show significant inhibition of the growth with MICs ≤ 64, and 256 μg/mL, respectively (**Figure 7**; **Supplementary Table S4**).

**Figure 8:**
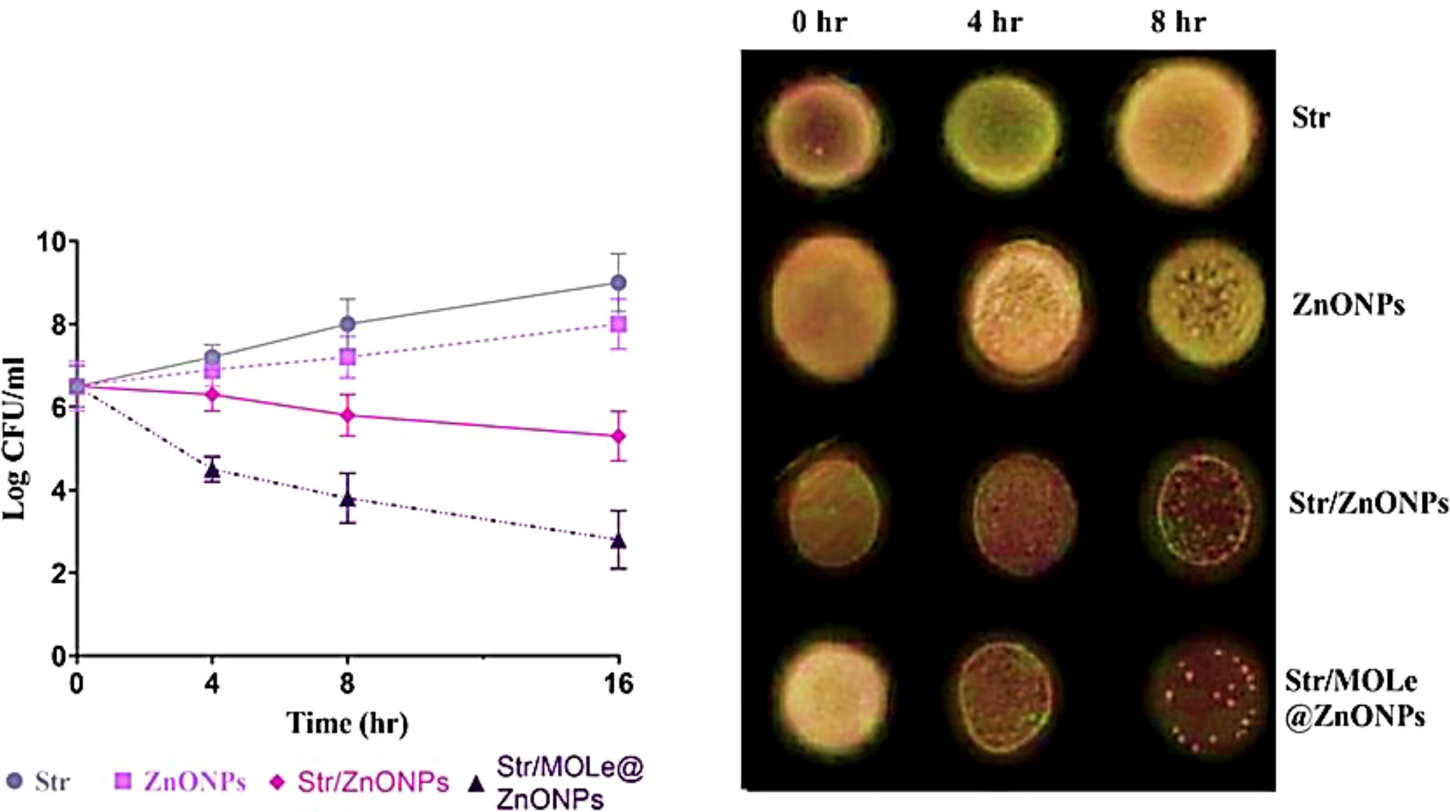
The time-kill curve and the bactericidal mechanism of ZnONPs, Str/ZnONPs and Str/MOLe@ZnONPs compared with Str at time point 0, 4, 8, 16 h on pandrug-resistant *E. faecalis* isolate of male origin (PDRM; isolate code 21). Each column indicates the mean ± SD from the three independent trials. Bacterial cultures from the MIC test were deposited on agar plates and incubated for 24 hours. The decrease in viable cell counts was observed at 0, 4, and 8 h.

The time-kill assay was assessed by culturing the *E. faecalis* isolate (exhibited PDR pattern; isolate code 21) under MIC- concentration of streptomycin, and Str/MOLe@ZnONPs and evaluating the number of viable bacteria (**Figure 8**). The viable bacteria count reduced from 10^6 CFU/mL to 10^4 CFU/mL after 4 hours of treatment with Str/MOLe@ZnONPs, and further dropped to 10^2 CFU/mL after 16 hours (**Figure 8**). The number of live bacteria reduced progressively to 10^5 CFU/mL after 16h of treatment with Str/ZnONPs. The findings showed that Str/MOLe@ZnONPs had bactericidal effects in a short period. On the other hand, both MOLe@ZnONPs and Str antibiotic alone didn`t show a significant reduction in the number of viable bacteria with a slight increase in the number of viable counts turned from zero point till 16 h incubation.

#### 3.4.2 Anti-biofilm activity of Str/MOLe@ZnONPs

**Figure 9:**
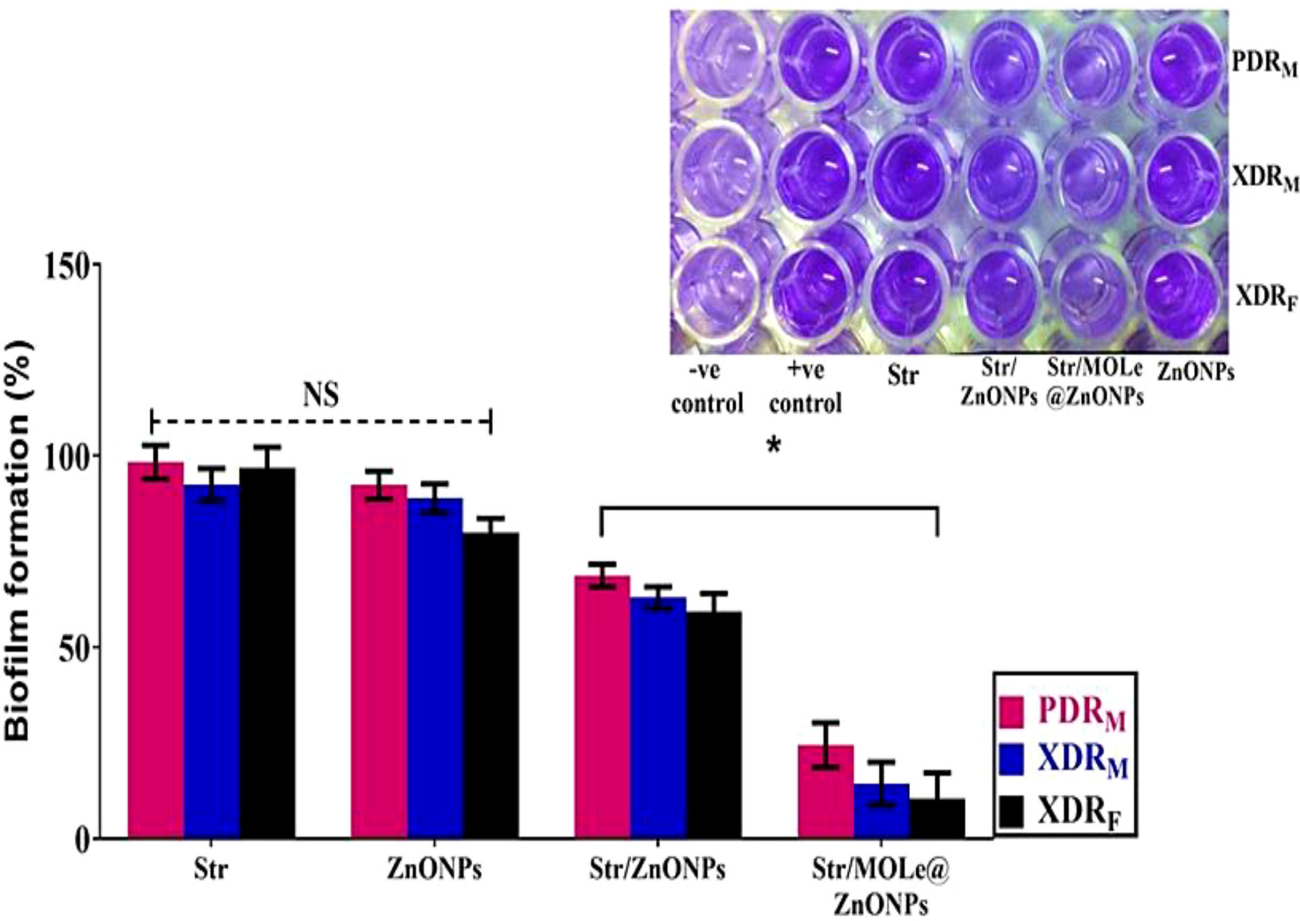
The biofilms formation by the crystal violet staining measuring at OD_600_ of extensively drug-resistant *E. fecalis* isolate of female origin (XDRF; isolate code 34), extensively drug-resistant *E. fecalis* isolate of male origin (XDRM; isolate code 32), and pandrug-resistant *E. fecalis* isolate of male origin (PDRM; isolate code 21) treated with ZnONPs, Str/ZnONPs and Str/MOLe@ZnONPs compared with Str. The columns demonstrate the mean ± SD of three independent experiments along with representative images. A greater intensity of the violet color indicates increased biofilm formation. “*” denotes statistically significant differences (P< 0.05), while “NS” indicates non-significant differences (P > 0.05) in comparison with the control sample.

CV staining showed that with sub-inhibitory concentration of Str/MOLe@ZnONPs, the biomass of biofilm was completely inhibited and the percentage biofilm formation ranged from 10-24% in treated isolates compared with the untreated (Positive controls)(*p*<0.05) (**Figure 9**). While, a moderate effect of Str/ZnONPs on biofilms were detected with a percentage ranged from 59-68% in treated biofilm mass (*p*<0.05). However, both of MOLe@ZnONPs, and streptomycin antibiotic alone didn`t show any detectable ability to reduce the biofilm mass with a range of 80–92%,and 92-98%, respectively (*p*>0.05).

**Figure 10:**
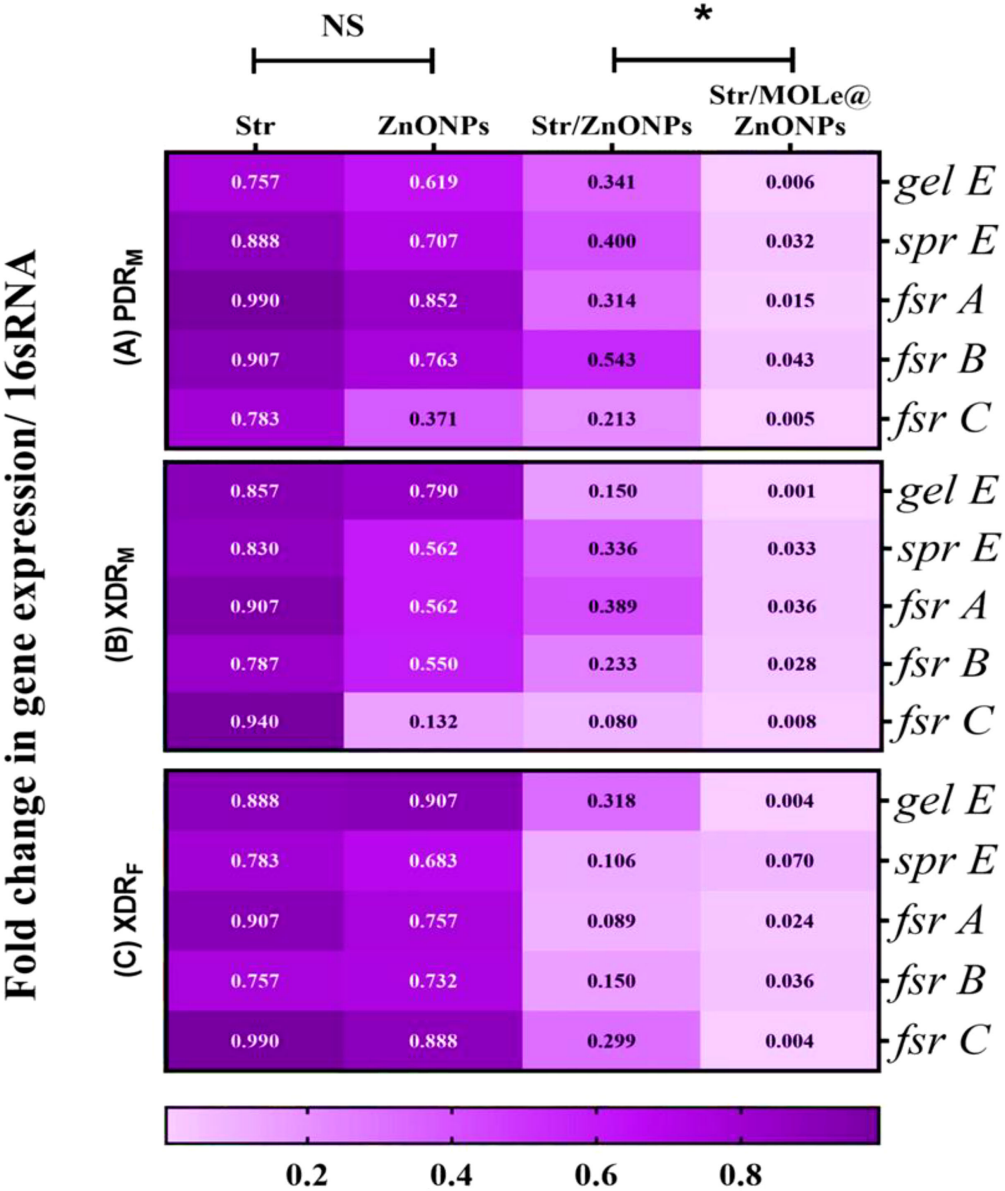
Heatmap of the relative gene expression of *gelE*, *sprE*, and *fsr (A,B,C)* upon treated with ZnONPs, Str/ZnONPs and Str/MOLe@ZnONPs compared with Str; extensively drug-resistant *E. fecalis* isolate of female origin (XDRF; isolate code 34), extensively drug-resistant *E. fecalis* isolate of male origin (XDRM; isolate code 32), and pandrug-resistant *E. fecalis* isolate of male origin (PDRM; isolate code 21). Gene expression levels were determined using the ΔΔCT method and shown as fold change. *16S rRNA* served as the endogenous control. The scale color indicates the level of down-regulation; associated genes with high levels of down-regulation are represented by the light mauve color, whereas genes with low level of down-regulation are represented by the deep mauve color. Each statistic represents the mean of three separate experiments. “*” indicates statistically significant differences (P < 0.05), while “NS” indicates non-significant changes (P > 0.05) in comparison with the control group.

To investigate the mechanism by which Str/MOLe@ZnONPs inhibit the biofilm formation of *E. faecalis*, real-time qRT-PCR was used to determine the expression of gelatinase (*gelE*), serine protease (*sprE*),and quorum-sensing *fsr* gene locus(*fsr;A,B,C*) virulence genes (**Figure 10**). The more vulnerable down-regulatory effect was detected in Str/MOLe@ZnONPs treated biofilm producer *E. faecalis* isolates as compared with the control group (*P*< 0.05). In which, the results showed that the fold change in *gelE, sprE*, and *fsr; A, B, and C* gene expression in PDR-treated *E. faecalis* [isolate code 21] were 0.06,0.032,0.015,0.043, and 0.05 respectively. Moreover, Str/ZnONPs showed a moderate, and significant down-regulatory effect on *gelE, sprE*, and *fsr; A, B, and C* genes expression compared with the control group *(P*< 0.05). On the other hand, both MOLe@ZnONPs, and streptomycin antibiotic alone didn`t significantly decrease the expression level of *gelE,sprE*, and *fsr; A, B, C* genes in treated *E. faecalis* isolates (*P*> 0.05).

## 4 Discussion

Historically, pets have been identified as a potential source of resistant microorganisms that might convey this resistance to humans (Kataoka et al., 2014; Jackson et al., 2009; Ossiprandi and Zerbini, 2015). Cats and dogs often carry antimicrobial drug-resistant enterococci which act as a source of these resistant microbes for humans. Enterococci often result in intricate urinary tract infections (Weese et al., 2011; Kajihara et al., 2015). The present study examined a 14% incidence rate of *E. faecalis* in urinary tract infections of domestic cats, with the majority of isolates coming from female cats, as previously reported (Oguttu et al., 2021). Drug-resistant *E. faecalis* strains have been on the rise, rendering many medications ineffective against infections caused by these bacteria (Oguttu et al., 2021). The research revealed a resistance level of 42.8% against aminoglycosides, which may be anticipated since Enterococci are naturally resistant to different doses of aminoglycosides, making them unsuitable for use as standalone treatments. The significant resistance to streptomycin at a high dose shown in recent investigations (Oguttu et al., 2021; Tumpa et al., 2022; Kristich et al., 2014).Worth noting, the increased resistance levels of 92.8% against ampicillin, compared with previously reported 41% (Oguttu et al., 2021). Previous investigation on the antimicrobial susceptibility of enterococci has confirmed the global rise of multi-drug resistant enterococci, especially to vancomycin (Werner et al., 2008). Despite the previously observed 28% resistance against vancomycin, alarming 64.2% of isolated *E. faecalis* species were resistant to vancomycin reported in this study.

*Enterococcus* species are inherently resistant and tolerant, and have the ability to quickly develop resistance to almost every antimicrobial agent used in clinical settings (Kristich et al., 2014).Therefore, we presented information on several drug resistance patterns (*n* = 12-pattern), with 64.2%, 28.5%, and 7.1% of the isolates showing MDR, XDR, and PDR categories, respectively. This resistance level was lower than what was previously mentioned (Oguttu et al., 2021; Tumpa et al., 2022).

Biofilms formation by Enterococci contribute to its virulence that allows the bacteria to irreversibly attached to urinary catheters (Costerton, 2001; Dautle et al., 2003), resistance to antibiotics (Mohamed et al., 2004).There was obvious association with higher prevalence of biofilm-forming strains and the sample source(urine and stool samples) (Hashem et al., 2017), and more than half of urinary tract infection isolated bacteria were biofilm producers (Akhter et al., 2014). Because of previously described superiority of using crystal Violet biofilm assay as a phenotypic detection of the biofilm-forming entercococcal clinical isolates (Sindhanai et al., 2016; Hassan et al., 2011). Our work demonstrated the using of already-established crystal violet assays to detect a higher frequency of biofilm forming *E. fecalis* than previously reported (Hashem et al., 2017), Another study team observed that 34% of their isolates developed powerful biofilms, 49% produced moderate biofilms, and 17% developed weak biofilms (Sindhanai et al., 2016).

The *gelE* gene encodes gelatinase activity, which is identified as the first crucial stage in the biofilm formation process. It serves as a trigger for bacterial surface adhesion and enhances the aggregation of microcolony cells (Hancock and Perego, 2004). *GelE* expression is positively regulated by the fsr locus controlled by quorum sensing (Nakayama et al., 2001; Qin et al., 2001).The fecal streptococci regulator *(fsr)* locus consists of three genes: *fsrA*, *fsrB*, and *fsrC*. It is a well-characterized quorum sensing system that controls *E. faecalis* biofilm formation and gene expression in reaction to high cell population densities (Miller and Bassler, 2001; Hancock and Perego, 2004). This locus is situated next to two genes that encode virulence factors: one encodes a gelatinase (*gelE*) and the other a serine protease (*sprE*). A prior work highlighted the significance of integrating patterns of the *gelE/fsrA, B* locus genes using genome screening and subsystem analysis to predict gelatinase activity linked with biofilm formation (Hashem et al., 2017).

All *E. faecalis* isolates were examined for the presence of biofilm-associated genes *fsr*, *gelE*, and *sprE*. The *gelE* gene was detected in 61.5% of biofilm-forming isolates. Previous studies have reported the presence of the *gelE* gene in 100%, 94%, 88%, and 92% of Enterococcus isolates (Hashem et al., 2017; Roberts et al., 2004; Mohamed and Murray, 2005; Sedgley et al., 2005). These reports, together with our findings, verify that *gelE* is crucial for biofilm development.

Potent biofilm-forming strains were found to possess the complete *fsrABC* gene locus, along with *gelE* and *sprE* genes. Conversely, a reduction in biofilm formation was associated with the absence of *gelE* and *sprE* genes. These findings align with previous research demonstrating that the absence of *fsr* and/or gelatinase activity leads to reduced biofilm development (Mohamed et al., 2004). Increased use of antimicrobials leads to serious the development of enterococci resistance. Besides, the high resistance of Enterococcus to aminoglycoside renders the use of this antibiotic as a single agent (García-Solache and Rice, 2019). Accordingly, investigation of new antimicrobial agents is an ultimate need (Cegelski et al., 2008; Lallo da Silva et al., 2019; Essawi et al., 2020; El-Demerdash et al., 2023a; Ebrahem et al., 2024).

Nanomaterials provide potential solutions that circumvent typical antibiotic resistance mechanisms (Wang et al., 2017; Abd El-Emam et al., 2023; Hashem et al., 2024; Saad et al., 2024). Zinc oxide nanoparticles (ZnO NPs) do not have any adverse effects on health status (Sruthi et al., 2018), they show antibacterial properties against both Gram-positive and Gram-negative bacteria (Raghunath and Perumal, 2017; Liu et al., 2009; Reddy et al., 2014; Guo et al., 2015). TEM examination was used to analyze the surface morphology of the nanoformulations established in the investigation. The study was conducted at a decreased magnification scale of 50 and 100 nm. TEM analysis revealed distinct morphological surfaces in the produced formulations, resulting in materials with diameters ranging from 70 nm for ZnONPs, 200 nm for MOLe@ZnONPs, and 250 nm for Str/MOLe@ZnONPs.However, the addition of individual or dual drug caused no significant difference in the structure or morphology of the nanoparticles other than increased size within the nanoparticles. The increase in the size of nanoparticles upon drug loading was a clear indication of the successful drug trapping within the nanoparticles (Guo and Sun, 2020).

The findings of our study align with those of Chaudhary et al. (2019), who also observed a hexagonal form in the manufactured ZnO nanoparticles utilizing *Aloe vera* peel extract (Chaudhary et al., 2019). In addition, Salam et al. (2014) conducted a study in which they generated zinc oxide nanoparticles using a leaf extract from *O. basilicum* L. var. *purpurascens* Benth.-Lamiaceae. The researchers discovered that the nanoparticles exhibited a hexagonal (wurtzite) form and had a size of less than 50 nm. Divya et al. (2013) documented the presence of both spherical and hexagonal morphologies in zinc oxide nanoparticles that were generated using *H. rosa-sinensis* as the precursor.

However, the drug-loaded nanoparticles have higher zeta potential negative values because of the basicity of drugs which imparts a negative charge to the nanoparticles. The agglomeration of ZnO observed in the SEM and TEM supported the zeta potential and PDI readings (Abrahamse et al., 2017).

Nanoparticles with a narrow size distribution (PDI < 0.5) are often desired for biomedical applications. The DLS size and PDI of the synthesized nanoformulations ranged from 60 nm to 274 nm (Krug and Wick, 2011). Particles with a ζ-potential value above ±30 mV are generally considered more stable (Ali et al., 2018).The decrease in the negative ζ-potential charge observed in this study may be attributed to the ionization of hydroxyl groups (–OH) in the capping moieties at alkaline pH (Nava et al., 2011). This reduced negative charge indicates the successful loading of Str and MOLe onto the nanoparticles, which was confirmed by FTIR analysis (Asgari et al., 2014; Sawant and Bamane, 2018; Kumar et al., 2014; Nabiyouni et al., 2011).

For targeted biofilm delivery, it is crucial to extend the presence of the medicine over the microbial surface. Furthermore, we demonstrated the antibacterial efficacy of Str/MOLe@ZnONPs, Str/ZnONPs, and MOLe@ZnONPs against XDR and PDR *E. faecalis* isolates. The strains chosen for further testing using the *in vitro* agar well diffusion technique, MIC, and MBC tests were those exhibiting a robust biofilm-forming characteristic and resistance to a minimum of 7 antimicrobial agents. The study found that the mixture of ZnONPs with Str and MOLe had notable anti-enterococcal effects, with a growth inhibition zone of up to 40mm, MICs of < 16 μg/mL, and a quick decline in viable bacteria to 102 CFU/mL after 16 hours. These findings are supported by previous studies that assessed the inhibitory action of Str/ZnONPs, such as aminoglycosides-meropenem is a promising technique that significantly altered the resistance profile of multidrug-resistant P. aeruginosa strains by reducing MICs (El-Telbany et al., 2022; Fadwa et al., 2021). Meanwhile, a previous report showed a bacteriostatic rate of zinc oxide nanoparticles against *E. faecalis* (Bohan, 2018), our study reported the MIC of the Str/MOLe@ZnONPswere higher than those found in previous studies that using curcumin green synthesis of ZnONPsfor *E. faecalis* (El-Kattan et al., 2022; Sangani et al., 2015; Shinde, 2015; Aflatoonian et al., 2017). It is noteworthy that the combination of Streptomycin and ZnONPs exhibited heightened antibacterial efficacy against *E. faecalis*. The primary focus of the current study is on the observation that *E. faecalis*, which exhibited resistance to streptomycin, displays a significant increase in susceptibility to the same antibiotic with co-administration with nanoparticles. This specific insight provides opportunities to address the present dilemma of antibiotic resistance and effectively battle antimicrobial infections via mitigation strategies (Ghaffar et al., 2022).

Combining metal oxide nanoparticles, such as zinc oxide nanoparticles (ZnONPs), with antibiotics like streptomycin (Str) offers a promising approach to enhance antibiotic efficacy and overcome bacterial resistance. ZnONPs can improve the delivery and effectiveness of Str by increasing its binding to bacterial cells, inhibiting efflux pump activity, and disrupting bacterial membranes (Kotrange et al., 2021). Additionally, ZnONPs can generate reactive oxygen species (ROS), which further enhances Str’s bactericidal effects.

Previous studies have demonstrated significant improvements in antibacterial activity when combining Str with nanoparticles, with efficacy increases of up to 87.5% reported (Nishanthi et al., 2019; Salar et al., 2015; Aremu et al., 2021). This synergistic approach holds potential for combating antibiotic resistance in both Gram-positive and Gram-negative bacteria (Raghunath and Perumal, 2017; Liu et al., 2009; Reddy et al., 2014; Guo et al., 2015).

In this study, we specifically selected sub-minimum inhibitory concentrations (sub-MICs) to investigate biofilm-related effects, as these concentrations enable the detection of subtle influences on biofilm formation without inhibiting bacterial growth. This approach is consistent with previous report, which has shown that antimicrobial sub-MIC concentrations can promote biofilm formation in *Staphylococcus aureus* without affecting bacterial growth (Elawady et al., 2024). Notably, we observed a substantial reduction in biofilm biomass was noticed after exposure to a sub-minimum inhibitory concentration (sub-MIC) of Str/MOLe@ZnONPs, where the percentage of biofilm formation ranged from 10-24%. In line with other studies, proved that ZnONPs enhanced antibiofilm activity via the ability of its small size to penetrate the biofilm matrix (Fadwa et al., 2021), interfered with biofilm integrity either by interrupting exopolysaccharide synthesis (Al-Wrafy et al., 2022),or disturbing the process of biofilm formation, causing it to break apart and decompose (Abdel-Halim et al., 2022; Masák et al., 2014). Following previous reports indicated a synergistic antibiofilm effect against *S. aureus* of combination between ZnONPs and tested antibiotics (Abdelghafar et al., 2022).

In this study, we focused on the role of the *gelE* gene, which encodes a gelatinase enzyme strongly associated with biofilm formation in *Enterococcus* species. Gelatinase plays a crucial role in degrading extracellular matrix components, facilitating bacterial attachment and aggregation—key steps in biofilm formation (Şchiopu et al., 2023). Additionally, gelatinase can influence the modulation of virulence factors and contribute to the persistence of biofilms in hostile environments. Our results showed a decrease in the expression of *gelE*, along with other virulence genes, including *sprE* (serine protease) and the quorum-sensing *fsr* gene locus (*fsrA*, *fsrB*, *fsrC*), following treatment with ZnONPs combined with Streptomycin (Str) and MOLe.

This modulation of *gelE* and other virulence genes under sub-MIC concentrations suggests that these agents may interfere with key biofilm-related mechanisms, offering a potential therapeutic strategy for biofilm-associated infections. Previous research suggests that suppression of *FSR* quorum sensing genes may have an impact as an alternative strategy for therapeutic anti-enterococcal drug development (Tang et al., 2015; Desouky et al., 2013; Singh and Nakayama, 2014; Shojima and Nakayama, 2014). Metal oxide NPs as ZnONPs acting as QS inhibitors are promising new alternative against antibiotic resistant Gram-negative bacteria (Hayat et al., 2019).The quorum-quenching action of ZnONPs leading to reduction of biofilm regulating gene expression, and lowering the virulence efficacy, and biofilm ability of *P. aeruginosa* (Ali et al., 2020; El-Telbany et al., 2022), *S.aureus* (Abdelghafar et al., 2022), and *C. violaceum* (Kamli et al., 2021).

## 5 Conclusion

This study demonstrates the potent antibacterial and antibiofilm activities of ZnONPs combined with Str and MOLe against multidrug-resistant *E. faecalis*, including pan-drug-resistant isolates. The observed anti-biofilm effects are likely due to the inhibition of gelatinase (*gelE*), serine protease (*sprE*), and quorum-sensing *fsr* genes. These findings, supported by our gene expression analysis using novel, specifically designed primers, suggest that ZnONPs in combination therapy with traditional antibiotics and MOLe could be a promising therapeutic option for combating enterococcal infections.

## Data Availability

The datasets presented in this study can be found in online repositories. The names of the repository/repositories and accession number(s) can be found in the article/**Supplementary Material**.
