# Supplementary material for: Hexagonal zinc oxide nanoparticles: a novel approach to combat multidrug-resistant Enterococcus faecalis biofilms in feline urinary tract infections
Source: Front Cell Infect Microbiol. 2025 Jan 24;14:1505469. doi: 10.3389/fcimb.2024.1505469 (PMC11802582; doi:10.3389/fcimb.2024.1505469)
Supplement: Supplementary file 2 [file Table2.docx]

**Hexagonal Zinc Oxide Nanoparticles: A Novel Approach to Combat Multidrug-Resistant *Enterococcus faecalis* Biofilms in Feline Urinary Tract Infections**

**Alaa H. Sewid^1, 2^**^^[[1]](#footnote-1)^♣^**, Mohamed Sharaf^3, 4^**^♣^**,Azza S. El-Demerdash^5^*****,Sherif M. Ragab^4^, Fatimah O. Al-Otibi^6^, Mohamed Taha Yassin^6^, and Chen-Guang Liu^3^**

^1^Department of Microbiology, Faculty of Veterinary Medicine, Zagazig University, Zagazig, Egypt

^2^Department of Forestry, Wildlife and Fisheries, Institute of Agriculture, University of Tennessee Knoxville, Tennessee, USA

**^3^**Department of Biochemistry and Molecular Biology, College of Marine Life Sciences, Ocean University of China, Qingdao, 266003, PR China

^4^Department of Biochemistry, Faculty of Agriculture, AL-Azhar University, Nasr City, Cairo 11651, Egypt

^5^Laboratory of Biotechnology, Department of Microbiology, Agricultural Research Center
 (ARC), Animal Health Research Institute (AHRI), Zagazig 44516, Egypt

^6^Department of Botany and Microbiology, College of Science, King Saud University, Riyadh 11451, Saudi Arabia

Table S1. Prevalence of *E. faecalis* isolates originated from female, and male pet cats.

| Source (No.) | Overall Prevalence of *E. faecalis*  No. (%) |
| --- | --- |
| Female (50) | 8(16%) |
| Male (50) | 6(12%) |
| Total (100) | 14(14%) |

Table S2. Antimicrobial resistance pattern of *E****.*** *faecalis* isolates originated from female, and male pet cats.

| **Antimicrobial agent** | **Male** | **Female** | **Total** |
| --- | --- | --- | --- |
| **Streptomycin** | 3(50%) | 3(37.5%) | 6(42.8%) |
| **Imipenem** | 3(50%) | 2(25%) | 5(35.7%) |
| **Meropenem** | 3(50%) | 2(25%) | 5(35.7%) |
| **Ciprofloxacin** | 1(16.6%) | 1(12.5%) | 2(14.2%) |
| **Levofloxacin** | 1(16.6%) | 1(12.5%) | 2(14.2%) |
| **Vancomycin** | 4(66.6%) | 5(62.5%) | 9(64.2%) |
| **Teicoplanin** | 6(100%) | 8(100%) | 14(100%) |
| **Tigecycline** | 5(83.3%) | 7(87.5%) | 12(85.7%) |
| **Linezolid** | 6(100%) | 8(100%) | 14(100%) |
| **Ampicillin** | 5(83.3%) | 8(100%) | 13(92.8%) |
| **Doxycycline** | 3(50%) | 5(62.5%) | 8(57.1%) |

**Table S3.** Antibiotypes, MAR-indices, detection of PDR, XDR, and MDR, biofilm formation, and virulence genes detection of *E. faecalis* isolates originated from female, and male pet cats. (Sheet Excel)

**Table S4.** MIC and MBC (µg/mL) of Str/MOLe@ZnONPs, Str/ZnONPs, MOLe@ZnONPs, and Str for XDRF (isolate code 34), XDRM (isolate code 32), and PDRM (isolate code 21) *E. faecalis* isolates

| *E. faecalis* isolates | **Str** | | **ZnONPs** | | **Str@ZnONPs** | | **Str/MOLe@ZnONPs** | |
| --- | --- | --- | --- | --- | --- | --- | --- | --- |
|  | MIC | MBC | MIC | MBC | MIC | MBC | MIC | MBC |
| **XDRF** | 128 | 256 | 16 | 32 | 8 | 16 | 4 | 8 |
| **XDRM** | 128 | 256 | 32 | 64 | 16 | 32 | 8 | 16 |
| **PDRM** | 256 | 512 | 64 | 128 | 32 | 64 | 16 | 32 |

MIC: Minimum inhibitory concentration, MBC: Minimum bactericidal concentration


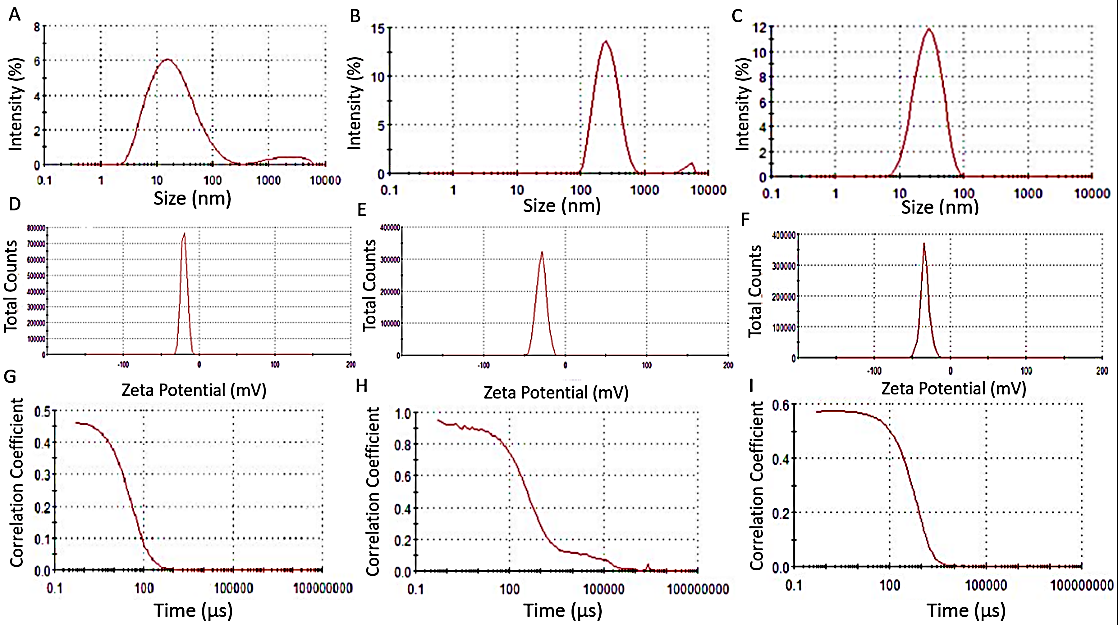


**FigureS1.**Hydrodynamic size, polydispersity index (PDI), ζ-potential and Row Coloration Data (RCD) of ZnONPs **(A, D and G)**, Str/ZnONPs **(B, E and H)**, and Str/MOLe@ZnONPs**(C, F and I)** .The numbers are given as mean standard deviation (SD) for ζ-potential (*n* = 3) and for particle size and PDI (*n* = 4).


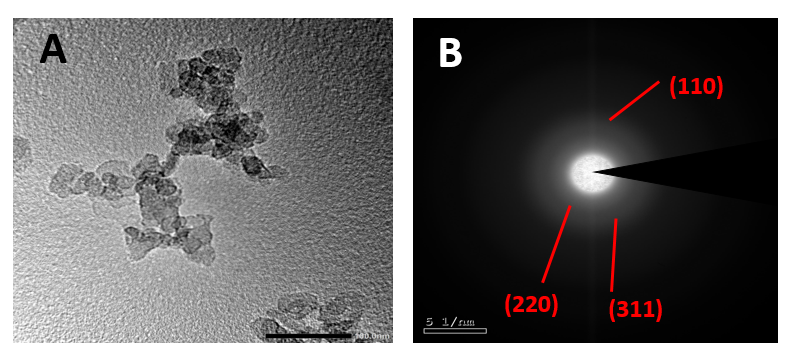


**FigureS2.**(A) High-resolution transmission electron microscopy (HTEM) and (B) selected area electron diffraction (SAED) of ZnONPo


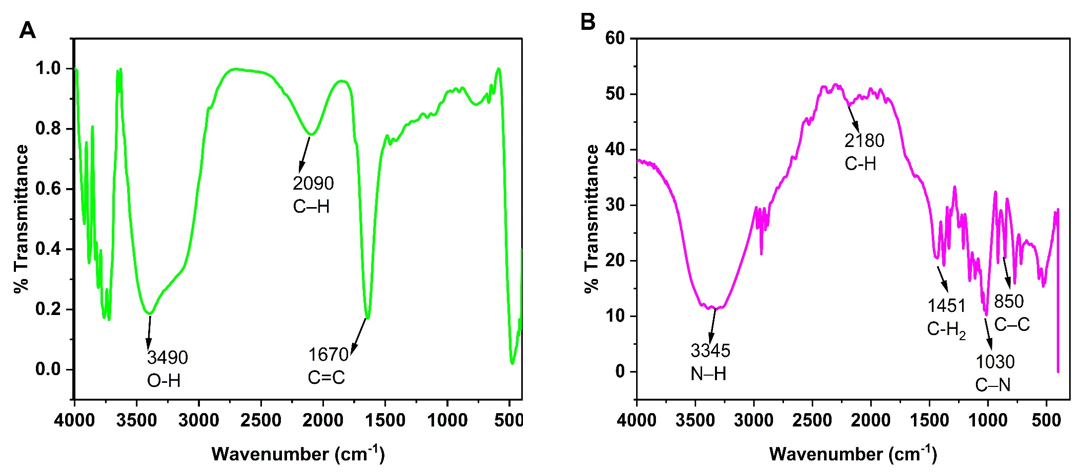


**FigureS3**. FTIR spectra analyzed of the synthesis of **(A)**MOLe, and **(B)** streptomycin (Str)

1. ^♣^These authors contributed equally to this work

   *Corresponding author: *E-mail address*;[dr.azzasalah@yahoo.com](mailto:dr.azzasalah@yahoo.com); drazza@ahri.gov.eg [↑](#footnote-ref-1)
